# Air Quality Change in Seoul, South Korea under COVID-19 Social Distancing: Focusing on PM_2.5_

**DOI:** 10.3390/ijerph17176208

**Published:** 2020-08-27

**Authors:** Beom-Soon Han, Kyeongjoo Park, Kyung-Hwan Kwak, Seung-Bu Park, Han-Gyul Jin, Sungju Moon, Jong-Won Kim, Jong-Jin Baik

**Affiliations:** 1 School of Earth and Environmental Sciences, Seoul National University, Seoul 08826, Korea; hanbs2001@snu.ac.kr (B.-S.H.); kjmon121@snu.ac.kr (K.P.); seung9@snu.ac.kr (S.-B.P.); hgjin@snu.ac.kr (H.-G.J.); sjmoon90@snu.ac.kr (S.M.); jwkim73@snu.ac.kr (J.-W.K.); jjbaik@snu.ac.kr (J.-J.B.); 2 School of Natural Resources and Environmental Science, Kangwon National University, Chuncheon 24341, Korea

**Keywords:** COVID-19, social distancing, PM_2.5_, urban air quality, Seoul, air quality monitoring station

## Abstract

Seoul, the most populous city in South Korea, has been practicing social distancing to slow down the spread of coronavirus disease 2019 (COVID-19). Fine particulate matter (PM_2.5_) and other air pollutants measured in Seoul over the two 30 day periods before and after the start of social distancing are analyzed to assess the change in air quality during the period of social distancing. The 30 day mean PM_2.5_ concentration decreased by 10.4% in 2020, which is contrasted with an average increase of 23.7% over the corresponding periods in the previous 5 years. The PM_2.5_ concentration decrease was city-wide and more prominent during daytime than at nighttime. The concentrations of carbon monoxide (CO) and nitrogen dioxide (NO_2_) decreased by 16.9% and 16.4%, respectively. These results show that social distancing, a weaker forcing toward reduced human activity than a strict lockdown, can help lower pollutant emissions. At the same time, synoptic conditions and the decrease in aerosol optical depth over the regions to the west of Seoul support that the change in Seoul’s air quality during the COVID-19 social distancing can be interpreted as having been affected by reductions in the long-range transport of air pollutants as well as local emission reductions.

## 1. Introduction

The rapid spread of coronavirus disease 2019 (COVID-19), now declared a pandemic by the World Health Organization (WHO), prompted the national and municipal governments around the world to take unprecedented measures, such as lockdowns, strict travel bans, and other restrictions on human activity. In South Korea, the first COVID-19 case was confirmed on 20 January 2020, and there was a dramatic rise in new cases, starting from 18 February 2020, following several clusters of mass infections around the country. On 29 February 2020, the term “social distancing” was first used at a press briefing conducted by the Korea Centers for Disease Control and Prevention (KCDC) in reference to a set of recommended practices including staying away from crowded spaces, avoiding non-essential travel, and keeping a safe interpersonal distance of at least 2 m.

The global responses to COVID-19, ranging from lockdowns to social distancing guidelines, offer a rare opportunity for assessing, through real-world events, the environmental scenarios conditioned by reductions in local air pollutant emissions. For this reason, scientists began looking into air pollution mitigation as a side-effect of the COVID-19 induced restrictions [1,2]. Clearer signs of PM_2.5_ and NO_2_ air quality improvements were seen at a city-scale rather than at a national-scale [3,4,5], suggesting that the reductions in air pollutant emissions were mainly concentrated in densely populated areas or areas with large industrial complexes. Large cities around the world reported considerable reductions in air pollutant emissions, particularly from traffic and industrial activities [6,7,8]. Reductions in primary air pollutant emissions under COVID-19 led to improved air quality in terms of decreased concentrations of carbon monoxide (CO), sulfur dioxide (SO_2_), nitrogen dioxide (NO_2_), and particulate matter with a diameter of 2.5 μm or less (PM_2.5_) in Brazil [9,10], China [11,12,13,14], India [15], and Italy [16]. Similar reductions in PM_2.5_ concentration in three Asian cities under COVID-19 and its positive effects on human health are reported in [17]. Adversely, such reductions may have contributed to the elevated ozone (O_3_) concentrations in Wuhan and cities in Southern Europe [18]. On the other hand, social restrictions might not lead to a significant difference in air quality in cities with low baseline concentrations of pollutants such as New York [19]. Owing to the apparent diversity in the air quality and COVID-19 situations among different regions, a specific case study that takes into account the unique conditions in each city can be helpful.

Seoul is the capital of South Korea with a population of approximately 10 million. Between 30 January to 29 March 2020, the cumulative total number of COVID-19 cases in Seoul rose from four to 410, over a 100-fold increase, in the span of 60 days. It is probable that, faced with the nation-wide rise in the number of new cases, the residents of Seoul cooperated with social distancing guidelines, particularly following the government announcement on 29 February 2020. Such a decline in human activity may have led to reductions in local air pollutant emissions. Note that this is not the first time Seoul has experienced an improvement in air quality in terms of decreased concentrations of primary pollutants owing to emission reductions from societal causes. During the economic recession of 1998, Seoul saw a 9 μg m^−3^ (13%) decrease in annual PM_10_ concentration compared to 1997 [20]. According to the National Air Pollutants Emission Service of Korea [21], as of 2017, the annual PM_2.5_ and PM_10_ emissions in Seoul, excluding resuspension sources, are largely comprised of construction and on-road traffic sectors. Therefore, the restrictions to human activity in Seoul by social distancing can still have a significant impact on the overall air quality in the city.

This study is a report on the air quality change in Seoul during the period of COVID-19 social distancing, with emphasis on PM_2.5_ concentration. Using air quality monitoring data, we first make comparisons between the periods before and after the start of social distancing in 2020, as well as comparisons against the climatological air quality in the past 5 years, and discuss the possible contributors to such changes in air quality.

## 2. Data and Methods

In this study, we recognize 29 February 2020 as the start of social distancing based on the government announcement made on that date. Accordingly, we define the periods of 30 days before and after the start of social distancing as pre-SD (from 30 January to 28 February 2020) and SD (from 29 February to 29 March 2020), respectively. Regarding the data from the past 5 years from 2015 to 2019, the same dates were compared to the pre-SD of 2020. For comparisons against SD of 2020, the 30 days from 1 to 30 March in the years 2015, 2017, 2018, and 2019 and those from 29 February to 29 March in the leap year (2016) were used.

**Figure 1:**
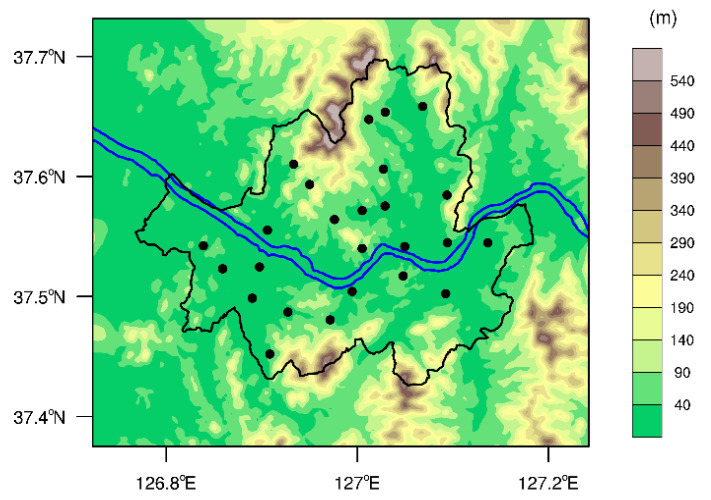
Terrain height of Seoul and surrounding areas. The black and blue solid lines represent the city limit of Seoul and the Han river, respectively. The 25 AQMSs are indicated by the black dots.

The hourly-averaged PM_2.5_, PM_10_, NO_2_, O_3_, CO, and SO_2_ concentrations measured at 25 air quality monitoring stations (AQMSs) located in each district of Seoul were provided by Korea Environment Corporation [22]. Figure 1 shows the locations of the AQMSs and terrain height of Seoul and surrounding areas. Note that these AQMSs are distinguished from roadside monitoring stations, and their locations tend to avoid being too close to major avenues and highways to capture representative measurements of urban air quality in Seoul. From the monitoring station data for the analysis periods in 2015–2020, a total of 1,263,418 pollutant concentration measurements were used in this study. This excludes the 32,582 missing values, which account for 2.5% of the total data.

Traffic volume data were acquired from the Seoul Transport Operation and Information Service [23] to estimate the reduction in human activity due to social distancing. Out of the 145 vehicle-counting sites, 53 sites without missing values during the 60 day periods corresponding to pre-SD and SD in 2020 were selected in this study.

To examine air quality in wider areas around Seoul and the transport of air pollutants, the aerosol optical depth (AOD) from the MERRA-2 data [24] produced by the National Aeronautics and Space Administration (NASA) [25] were analyzed. The spatial and temporal resolutions of the MERRA-2 data are 0.5° × 0.625° and 3 h, respectively. In addition, the zonal and meridional wind components and geopotential height at the 900 hPa level from the ERA5 data operated by the European Centre for Medium-Range Weather Forecasts (ECMWF) [26] were used to investigate meteorological conditions before and after the start of social distancing. We analyzed the meteorological variables at the 900 hPa level to illustrate the transport of air pollutants by air flows in the lower atmosphere. The ERA5 data have a spatial resolution of 0.25° × 0.25° and a temporal resolution of 1 h.

## 3. Results and Discussion

**Figure 2:**
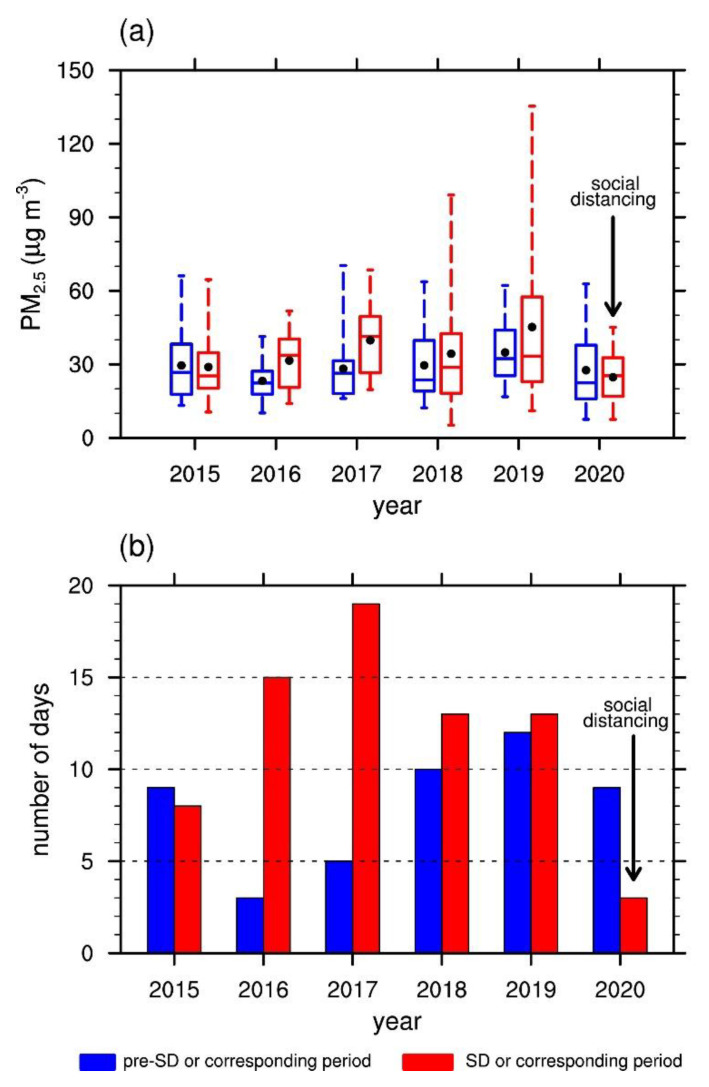
(**a**) Box plot of the daily mean PM_2.5_ concentrations in the 30 days of pre-SD in 2020 and the corresponding periods in 2015–2019 (blue) and SD in 2020 and the corresponding periods in 2015–2019 (red). The box indicates the lower and upper quartiles. The center line and black dot inside each box represent the median and 30 day mean values, respectively. The whiskers above and below each box indicate the maximum and minimum values, respectively. (**b**) Numbers of days with “high” levels of PM_2.5_ concentration (>35.0 μg m^−3^) out of the 30 days of pre-SD in 2020 and the corresponding periods in 2015–2019 (blue) and SD in 2020 and the corresponding periods in 2015–2019 (red).

The daily mean PM_2.5_ concentration averaged over all stations in Seoul decreased by 10.4% from pre-SD to SD (Table 1). This is a notable decrease compared to the 23.7% increase on average over the same time period in the years 2015 through to 2019. Figure 2a shows that, from pre-SD to SD, the daily mean PM_2.5_ concentration experienced decreases in the maximum and the upper quartile. The difference between the upper and lower quartiles also decreased, indicating a smaller variability during SD of 2020. On the contrary, the upper quartiles of the daily mean PM_2.5_ concentrations in the previous years from 2016 and onward increased over the same time periods.

The reduction in variability appears to be related to the number of days with pollution levels reaching “high” in Figure 2b. The “high” days in this study are defined as the days with the daily mean PM_2.5_ concentration exceeding 35.0 μg m^−3^ in alignment with the guidelines set by the Korean government. Out of the 30 days of pre-SD and SD, the number of “high” days went down from 9 to 3, a dramatic decrease not found in the previous years. In fact, the years since 2016 through 2019 saw increasing trends in the number of “high” days over the same time period (Figure 2b). This appears to be a clear indication that social distancing exerted a downward pressure on air pollution levels.

By comparison, there have been reports of substantial decreases in PM_2.5_ concentrations due to reduced human activity related to COVID-19 (e.g., Collivignarelli et al. [16]; Kerimray et al. [7]; Li et al. [13]; Lian et al. [4]; Sharma et al. [15]; Zangari et al. [19]). For example, Wuhan, which was subjected to a series of strict lockdown orders, saw a 36.9% decrease in PM_2.5_ concentration [4]. Milan, another city hard-hit by COVID-19, experienced a decrease of 47.4% in PM_2.5_ concentration during a lockdown [16]. Unlike other major cities around the world, a complete city-wide lockdown was never imposed in Seoul, and social distancing was announced only as a recommendation. It is surprising that even this comparatively weaker forcing toward reduced human activity might have resulted in such noticeable improvements in the PM_2.5_ air quality in Seoul.

**Figure 3:**
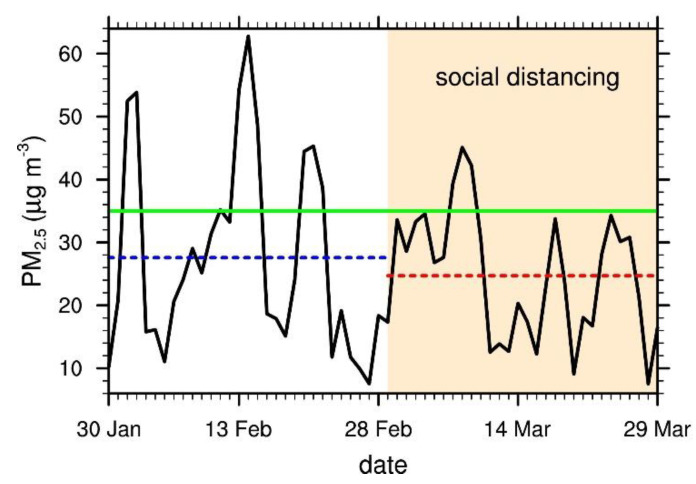
Time series of the daily mean PM_2.5_ concentration during pre-SD and SD in 2020. The horizontal dashed lines indicate the 30 day mean values for pre-SD (blue) and SD (red), respectively. The green horizontal solid line marks the threshold level of PM_2.5_ concentration (35.0 μg m^−3^, “high” if >35.0 μg m^−3^). The period of social distancing (SD) is indicated by the shaded area.

Figure 3 shows that major peaks in PM_2.5_ concentration appeared three times during each 30 day period: 2, 14, and 21 February during pre-SD, and 8, 18, and 24 March during SD. These peak PM_2.5_ concentrations can be attributed to factors other than local emission such as meteorological conditions associated with stagnant air in the region or the long-range transport of pollutants. That the PM_2.5_ concentration peaks reached higher levels pre-SD than during SD, with smaller fluctuations during SD, indicates that those other factors had a stronger influence in pre-SD than in SD.

To take a closer look at the effects of social distancing on PM_2.5_ concentration, workday–holiday comparisons are also considered. In this study, weekends and national holidays are classified as the holidays and the rest of the days are classified as the workdays. The daily mean PM_2.5_ concentrations averaged over the workdays were 25.0 μg m^−3^ and 25.5 μg m^−3^ in pre-SD and SD, respectively. In contrast, the holiday average saw a dramatic decrease from 34.8 μg m^−3^ in pre-SD to 23.2 μg m^−3^ in SD. Even considering the possibility that the results may have been skewed by a few high pollution events that coincidentally happened to occur on holidays during pre-SD, there were sizable decreases in holiday PM_2.5_ concentrations, which must at least partly reflect the suppressed emission due to social distancing during SD. Traffic data show an 8.4% decrease in the number of vehicles per day from pre-SD to SD on holidays compared to a 3.0% decrease on workdays.

**Figure 4:**
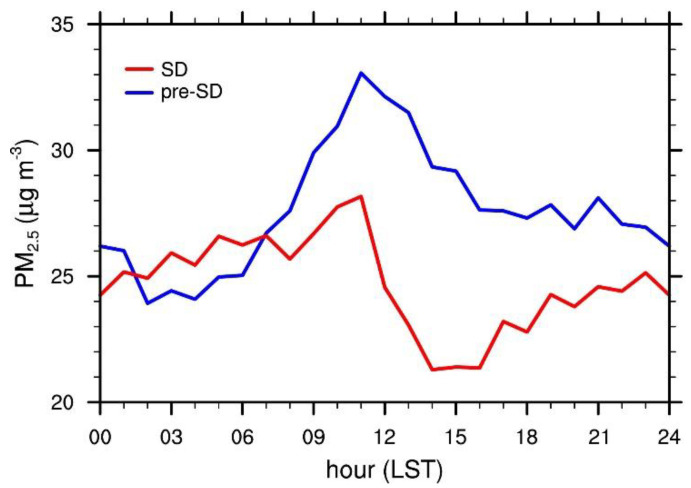
Diurnal variations of the 30 day mean PM_2.5_ concentrations averaged over all stations for pre-SD and SD.

The 30 day mean diurnal variations in the PM_2.5_ concentrations in Figure 4 also confirm that, overall, the PM_2.5_ concentrations were lower during SD than pre-SD. The maximum concentrations occur at the same time of the day (11 LST) for both pre-SD and SD, which can be attributed to the increased local emissions during the morning rush hour. The difference in PM_2.5_ concentration between SD and pre-SD reaches its peak at 13 LST during the hours when human activity levels are high, but is smaller during nighttime when human activity levels are thought to be low, even in the absence of social distancing. This might be an indication that, compared to nighttime, social distancing had a stronger influence on PM_2.5_ concentrations during high human activity hours.

Although the lower levels of PM_2.5_ concentration during SD can be explained in part by the reduced emissions due to social distancing, there may also be other complexities involved, such as variations in local weather conditions and the long-range transport of PM_2.5_. Further analyses of these variations may help explain the unexpected results exhibited by the diurnal variations such as the 4 h long window in early morning from 2 LST to 6 LST when SD had higher PM_2.5_ concentrations than pre-SD. The diurnal variation in PM_2.5_ concentration for pre-SD also exhibits a somewhat unusual downward-sloped tail end, where the variation is typically known to have an uptick as midnight approaches.

**Figure 5:**
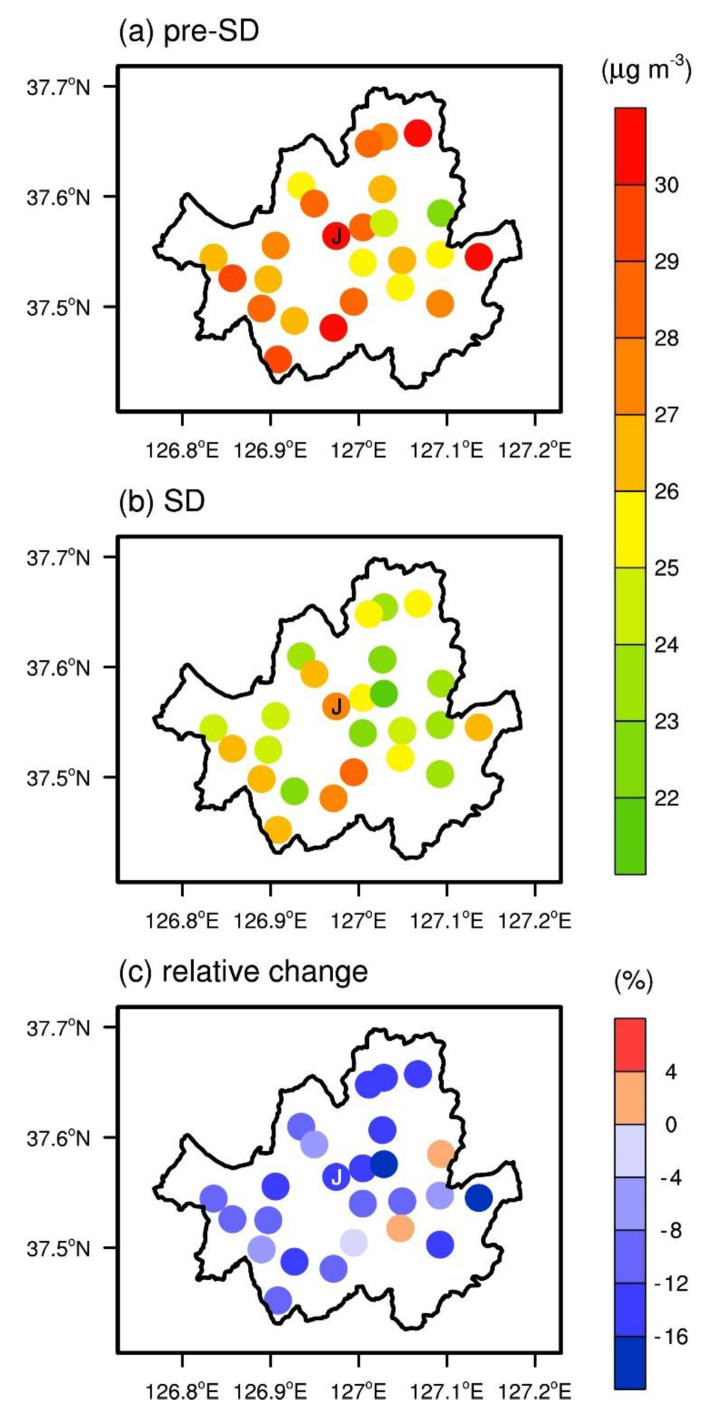
Spatial variations in the 30 day mean PM_2.5_ concentration for (**a**) pre-SD and (**b**) SD, and (**c**) relative change in the 30 day mean PM_2.5_ concentration from pre-SD to SD. The AQMS in Jung-gu is indicated by J.

The spatial distributions of PM_2.5_ concentrations in Seoul show that there was an across-the-board decrease from pre-SD to SD in almost all stations (Figure 5). In particular, the locations of the top 10 monitoring stations in terms of the magnitude of PM_2.5_ concentration decrease are clustered among the northern and central stations, including the station in Jung-gu, the traditional city center of Seoul. Empirical correlation coefficients (*R*) between PM_2.5_ concentrations at the Jung-gu station and other stations were calculated for quantitative analysis. From pre-SD to SD, the number of stations with which the Jung-gu station had empirical correlation coefficients exceeding 0.950 decreased from 19 to 4. Although other factors, such as meteorological conditions, can affect the empirical correlation coefficients, the change in the empirical correlation coefficient from pre-SD to SD implies that, to some extent, there were differences among the localities in responding to the social distancing guidelines during SD, leading to the spatial disparities in the changes in local emission.

**Figure 6:**
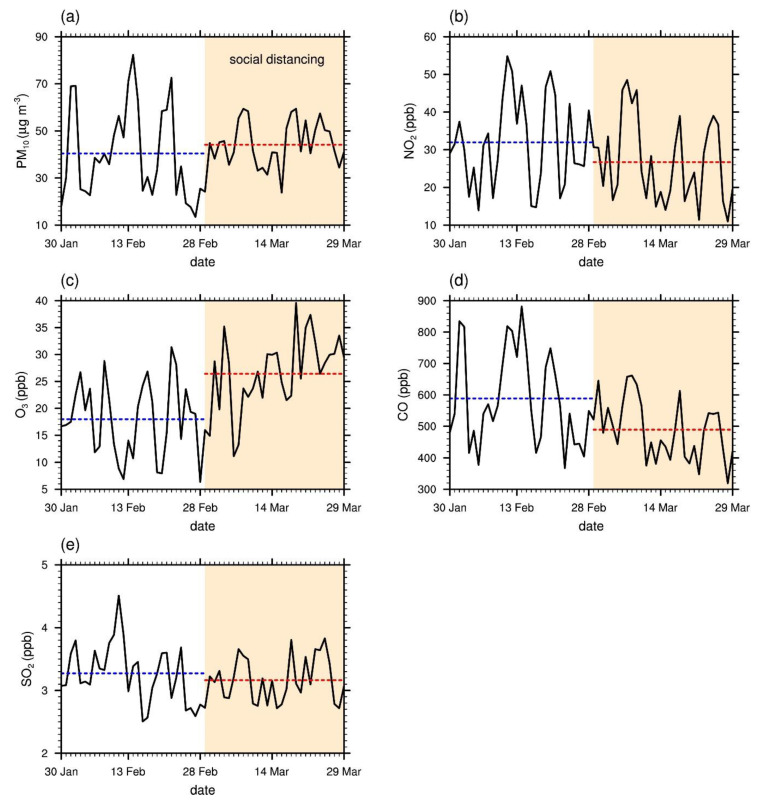
Time series of the daily mean values of (**a**) PM_10_, (**b**) NO_2_, (**c**) O_3_, (**d**) CO, and (**e**) SO_2_ concentrations during pre-SD and SD in 2020. The horizontal dashed lines indicate the 30 day mean values for pre-SD (blue) and SD (red), respectively. The period of social distancing (SD) is indicated by the shaded area.

Beyond PM_2.5_, the concentrations of other pollutants, PM_10_, NO_2_, O_3_, CO, and SO_2_, were examined for changes from pre-SD to SD. The daily mean concentrations of CO (Figure 6d), NO_2_ (Figure 6b), and, to a lesser extent, SO_2_ (Figure 6e) decreased on average by 16.9%, 16.4%, and 3.3%, respectively (Table 1). Furthermore, clear downward trends are seen in the time series for NO_2_ and CO but not so much for SO_2_. It is speculated that, unlike complete lockdowns, social distancing in Seoul mainly had effects on commuting and daytime social activities rather than industries, leading to a decreased level of traffic. Note that NO_2_ and CO are known to have direct links to vehicle emissions, whereas SO_2_ emission is largely associated with industrial activities [4]. According to the National Air Pollutants Emission Service of Korea [21], Seoul does not have a significant local source of SO_2_. That the relative decreases in NO_2_ and CO were larger than that in SO_2_, therefore, indicates reductions in local emissions due to decreased traffic volume as more people began following the social distancing guidelines over time since the initial announcement on 29 February 2020. The 30 day mean traffic volume in Seoul showed a 5.8% decrease from pre-SD to SD. The large increase in O_3_ concentration (47.0%) is also expected due to the less active O_3_ titration by NO as well as seasonal effects with the days getting longer into spring. This was also observed in other cities that experienced reduced levels of human activity due to COVID-19 (e.g., Collivignarelli et al. [16]; Kerimray et al. [7]; Li et al. [13]; Lian et al. [4]; Sicard et al. [18]).

PM_2.5_ shows a high empirical correlation with CO (*R* = 0.754). Since CO is more closely linked to local air pollutant emissions, this suggests that the decrease in PM_2.5_ concentration during SD can mainly be attributed to changes in local emissions [27,28]. The time series for PM_10_ in Figure 6a shows a high empirical correlation (*R* = 0.893) with that of PM_2.5_ in Figure 3. Similar to PM_2.5_, the daily mean PM_10_ concentrations exhibit a smaller variation during SD than during pre-SD. Although there is an increase (9.1%; Table 1) in the 30 day mean PM_10_ concentration, this is in the context of an even larger average increase (12.3%; Table 1) over the same time periods in the past 5 years. The ratio between the 30 day mean PM_2.5_ and PM_10_ concentrations decreased from 0.68 in pre-SD to 0.56 in SD. Although the ratio between PM_2.5_ and PM_10_ concentrations is affected by many factors, such as weather conditions and long-range transport of particulate matter, local emissions of anthropogenic pollutants tend to raise this ratio [29,30,31]. Therefore, the decrease in the ratio between PM_2.5_ and PM_10_ concentrations from pre-SD to SD can be explained in part by reduced anthropogenic emissions due to social distancing.

**Figure 7:**
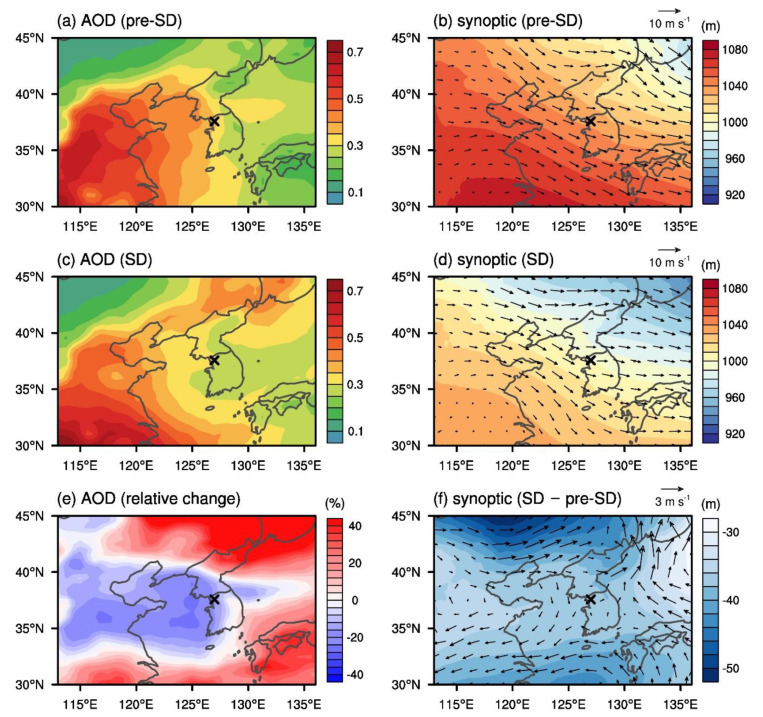
The 30 day mean AOD for (**a**) pre-SD and (**c**) SD, and (**e**) relative change in the 30 day mean AOD from pre-SD to SD. The 30 day mean geopotential height and wind vector at the 900 hPa level for (**b**) pre-SD and (**d**) SD, and (**f**) differences in the 30 day mean geopotential height and wind vector at the 900 hPa level from pre-SD to SD. The cross symbol indicates the location of Seoul.

Since the change in the air quality of Seoul may be associated with the air quality of its neighboring regions, the AOD field over the broad region encompassing the Korean Peninsula was analyzed. Note that the PM_2.5_ concentrations at the surface level may not have direct relations with the AOD distributions, which is a column integrated index that also includes information about aerosols at the upper levels as well as at the surface level. The 30 day mean AOD and the synoptic patterns at the 900 hPa level for pre-SD and SD are shown in Figure 7. The 30 day mean AOD for pre-SD has a steep eastward decline toward the Korean Peninsula. The AOD over Seoul (0.368) is only slightly higher than those over the other regions of South Korea along the same longitude despite Seoul being much more urbanized than other regions. This implies that the AOD over Seoul during pre-SD is partially explained by the transport of the aerosols with external origins. Figure 7b shows the average northwesterly flow over the Yellow Sea transporting aerosols from the west during pre-SD. During SD, the 30 day mean AOD decreased over parts of China, the Yellow Sea, and the west coast of the Korean Peninsula (~33–41° N, ~113–128° E) (Figure 7c,e). Across these regions, the circulation difference from pre-SD is anti-cyclonic, and there exists a small relative divergence (Figure 7f), suggesting that the eastward aerosol transport to Seoul may have been slightly reduced during SD. The 30 day mean AOD over Seoul decreased by 16.5% from pre-SD to SD. According to the Korea Ministry of Environment (2019), the annual contribution to the PM_2.5_ concentration in Seoul during 2013–2017 was comprised of domestic sources (45%) and transboundary transport from China (34%), Japan (1%), and other countries (20%) [32]. In another study, the long-range transport of pollutants was estimated to have raised PM_10_, NO_2_, CO, and SO_2_ concentrations from 2001 to 2014 in Seoul by 21%, 9%, 6%, and 14%, respectively [33]. Considering the above analyses, our results imply that the reduction in the eastward transport of pollutants to Seoul, in addition to the reduced local emissions in Seoul, contributed to the decrease in PM_2.5_ concentration during SD.

## 4. Conclusions

PM_2.5_ and the five other air pollutant concentrations, measured in Seoul over the 30 days before and after the start of social distancing, were analyzed to illustrate the impact of COVID-19 social distancing on the air quality of Seoul. The 30 day mean PM_2.5_ concentration averaged over all stations in Seoul decreased by 10.4% since the start of social distancing, while it increased by an average of 23.7% over the same time periods in the years 2015–2019. Between pre-SD and SD, there was a dramatic drop in the number of “high” days exceeding the PM_2.5_ concentration of 35.0 μg m^−3^ from 9 to 3 days out 30, while this number went up over the same time periods every year from 2016 to 2019. This study shows that social distancing, a much weaker response to COVID-19 than a lockdown, can lead to decreases in air pollutant concentrations in megacities. The diurnal variations in PM_2.5_ concentration during pre-SD and SD confirm that PM_2.5_ concentration especially decreased during high human activity hours. The spatial distribution of the PM_2.5_ concentration difference between pre-SD and SD illustrates that the decrease in PM_2.5_ concentration occurred throughout the whole city, except for a few stations. Other pollutants such as NO_2_, CO, and SO_2_ also experienced decreases in concentration, which are partly attributed to the reduced local emissions during SD. On the other hand, O_3_ concentration increased by 47.0% because of the less active O_3_ titration by NO. The reductions in air pollutants from pre-SD to SD are confirmed again through the analysis of the AOD. The AOD over Seoul decreased by 16.5% from pre-SD to SD, and this decrease can be attributed to the change in synoptic circulation and long-range transport during SD and the reductions in local emissions under social distancing.

Further investigation is needed to better understand the spatial variability of the PM_2.5_ concentration decrease, which may have been affected by a variety of influencing factors, such as topography and sociological characteristics. In addition to the impact of emission reduction on air quality, the impact of meteorological conditions also deserves an investigation. Estimating the relative contributions of local emissions and long-range transport to the air quality change under social distancing is a challenging problem because of the difficulties in properly separating the two contributors using measurement data alone. Numerical simulations of air quality using a high-resolution chemistry-coupled atmospheric model can help estimate the relative contributions of local emissions and long-range transport, which deserves investigation.

## Figures and Tables

**Table 1:** Thirty day mean concentrations of air pollutants for pre-SD and SD in 2020 and the corresponding periods in 2015–2019, the relative changes from pre-SD to SD in 2020 and the corresponding periods in 2015–2019, and the relative changes from the periods corresponding to SD in 2015–2019 to SD in 2020.

Pollutant/Year	Pre-SD *	SD *	Relative Change (%) Pre-SD * vs. SD *	Relative Change (%) for SD * 2015–2019 vs. 2020
PM_2.5_ (μg m^−3^)				
2020	27.6	24.7	−10.4	−31.2
2015–2019	29.1	35.9	23.7	
PM_10_ (μg m^−3^)				
2020	40.4	44.1	9.1	−29.9
2015–2019	56.0	62.9	12.3	
NO_2_ (ppbv)				
2020	32.0	26.7	−16.4	−26.9
2015–2019	34.9	36.5	4.7	
O_3_ (ppbv)				
2020	18.0	26.4	47.0	5.7
2015–2019	17.5	25.0	42.5	
CO (ppbv)				
2020	588.3	489.1	−16.9	−16.1
2015–2019	633.4	583.3	−7.9	
SO_2_ (ppbv)				
2020	3.3	3.2	−3.3	−41.5
2015–2019	5.6	5.4	−2.5	

* For 2015–2019, the periods corresponding to pre-SD and SD in 2020 were used for the calculation.
